# Rethinking the “Diseases of Affluence” Paradigm: Global Patterns of Nutritional Risks in Relation to Economic Development

**DOI:** 10.1371/journal.pmed.0020133

**Published:** 2005-05-03

**Authors:** Majid Ezzati, Stephen Vander Hoorn, Carlene M. M Lawes, Rachel Leach, W. Philip T James, Alan D Lopez, Anthony Rodgers, Christopher J. L Murray

**Affiliations:** **1**Harvard School of Public Health, BostonMassachusettsUnited States of America; **2**Clinical Trials Research UnitUniversity of AucklandNew Zealand; **3**International Obesity Task ForceLondonUnited Kingdom; **4**School of Population Health, University of QueenslandBrisbaneAustralia; University of California at San FranciscoUnited States of America

## Abstract

**Background:**

Cardiovascular diseases and their nutritional risk factors—including overweight and obesity, elevated blood pressure, and cholesterol—are among the leading causes of global mortality and morbidity, and have been predicted to rise with economic development.

**Methods and Findings:**

We examined age-standardized mean population levels of body mass index (BMI), systolic blood pressure, and total cholesterol in relation to national income, food share of household expenditure, and urbanization in a cross-country analysis. Data were from a total of over 100 countries and were obtained from systematic reviews of published literature, and from national and international health agencies.

BMI and cholesterol increased rapidly in relation to national income, then flattened, and eventually declined. BMI increased most rapidly until an income of about I$5,000 (international dollars) and peaked at about I$12,500 for females and I$17,000 for males. Cholesterol's point of inflection and peak were at higher income levels than those of BMI (about I$8,000 and I$18,000, respectively). There was an inverse relationship between BMI/cholesterol and the food share of household expenditure, and a positive relationship with proportion of population in urban areas. Mean population blood pressure was not correlated or only weakly correlated with the economic factors considered, or with cholesterol and BMI.

**Conclusions:**

When considered together with evidence on shifts in income–risk relationships within developed countries, the results indicate that cardiovascular disease risks are expected to systematically shift to low-income and middle-income countries and, together with the persistent burden of infectious diseases, further increase global health inequalities. Preventing obesity should be a priority from early stages of economic development, accompanied by population-level and personal interventions for blood pressure and cholesterol.

## Abstract 2

Cardiovascular diseases, traditionally thought of as diseases of affluence, are likely to become a substantial public health in low-income and middle-income countries.

## Introduction

**Figure 1:**
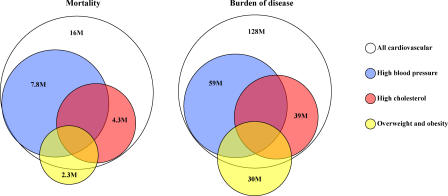
Global Mortality and Burden of Disease Attributable to Cardiovascular Diseases and Their Major Risk Factors for People 30 y of Age and Older The size of each circle is proportional to the number of deaths (left) or burden of disease (right; measured in disability-adjusted life years) (in millions). Overweight and obesity affect non-cardiovascular diseases, including diabetes, endometrial and colon cancers, post-menopausal breast cancer, and osteoarthritis, shown as the portions of yellow circles that fall outside the cardiovascular disease circle [57]. The mortality estimates exclude osteoarthritis, which results in morbidity but not direct deaths. Disease burden does include nonfatal health outcomes associated with diabetes and osteoarthritis (hence the larger size of the circle for overweight and obesity relative to those for blood pressure and cholesterol). Source: re-analysis of data from Ezzati et al. [57,58].

Cardiovascular diseases and their nutritional risk factors are among the leading causes of mortality and morbidity globally (Figure 1), and have been predicted to rise over the next few decades [1–3]. Aging of the world's population is a key driver of the expected increase, because cardiovascular disease rates tend to increase with age. In addition to this demographic change, an epidemiological change that involves increases in age-specific rates of cardiovascular diseases in developing countries has also been predicted in some analyses [4]. This epidemiological change is a corollary to a predicted population-wide rise in cardiovascular disease risk factors including obesity, blood pressure, cholesterol, and tobacco use with increasing income, originally referred to as the “diseases of affluence” or “Western disease” paradigm [5,6]. A number of challenges have been made to the diseases of affluence paradigm. For example, it has been observed that cardiovascular diseases and some of their risk factors (e.g., smoking) may decline once they have peaked [7]. It has also been documented that within upper-middle-income and high-income countries, cardiovascular diseases and risk factors are increasingly concentrated among the lowest socioeconomic groups [8–11].

Despite these challenges in specific populations, at the global level, predictions about rising levels of cardiovascular risk factors with economic development continue to be made [2,12,13]. The global health aspect of the diseases of affluence paradigm is particularly important because it implies that a large proportion of the world's population, who live in middle-income countries, will soon face both aging populations and rising age-specific cardiovascular disease rates, and will require increasing focus on policies and interventions to reduce the resulting disease burden [13]. Yet the timing of initiating interventions during a society's economic development and the specific form of the required interventions have not been addressed based on systematic analyses of risk factor and disease profiles. The diseases of affluence paradigm also implies that cardiovascular disease risk factors are not urgent public health concerns for low-income populations. We systematically examined the population-level relationships between three leading nutritional cardiovascular risk factors—overweight and obesity, elevated blood pressure, and cholesterol—and three economic variables using data for over 100 countries. Analysis of multiple nutritional risks shows more complex economic–epidemiological patterns than those predicted by simple descriptions such as the “diseases of affluence” or “Western disease” paradigms. More importantly, focusing on multiple risk factors helps identify specific intervention and policy options and priorities, with implications for societies at various levels of economic development.

## Methods

We examined the cross-sectional relationship between mean population blood pressure, cholesterol, and body mass index (BMI) and three socioeconomic variables: national income, average share of household expenditure spent on food, and proportion of population in urban areas. Blood pressure, cholesterol, and BMI are well-established cardiovascular risk factors and provide aggregate indicators of more complex dietary patterns (e.g., caloric intake, and consumption of salt, fats of different composition, and fruits and vegetables) and physical activity. Further, there are more comparable data from population-based health and nutrition surveys on these physiological indicators than on dietary patterns and physical activity, because these indicators can be more easily defined in a consistent manner and measured using standard techniques. National income is the commonly used indicator for a society's material well-being. The share of household expenditure spent on food is a measure of how household economic resources may constrain food purchase. If food forms a large proportion of total household expenditure, households may limit the total amount of food consumed, which should result in lower obesity and possibly lower levels of other risk factors if they are affected by overconsumption, or households may switch to less expensive but lower quality foods that increase various nutritional risk factors (e.g., higher salt, leading to higher blood pressure, or higher sugar and fat, leading to higher obesity) [14]. The proportion of the population living in urban areas is a proxy indicator of a number of environmental and lifestyle variables, such as physical activity in occupational and transportation domains, and of access to specific food types. For example, people living in rural areas often have higher levels of physical activity, reflecting their agricultural occupations and the need to walk longer distances for day-to-day activities [15]. Similarly, rural and urban populations may have differential access to various food types, possibly with seasonal variations. Urban populations are also likely to have higher access to screening and treatment for risks such as high blood pressure and cholesterol.

### Data Sources

Data sources for risk factors and economic indicators are provided in Table 1 (see Table S1 for list of countries). Systolic blood pressure (SBP), cholesterol, and BMI were age-standardized so that results could be compared across populations with different age structures. We used the World Health Organization (WHO) standard population [16] because it is a better representation of current population age structures than older standard populations (e.g., SEGI). The data used in the analysis were collected by researchers using different instruments. Strict criteria for study selection were applied to ensure methodological rigor and representativeness [17–19]. There were, nonetheless, differences among studies in a number of dimensions: sample size, national (versus sub-national) representativeness, year of the study, and age groups included. When restricted to only large-scale, nationally representative studies, the analysis resulted in associations very similar to those presented using the entire dataset; heterogeneity decreased after excluding all countries that did not fulfill these more stringent criteria.

**Table 1:**
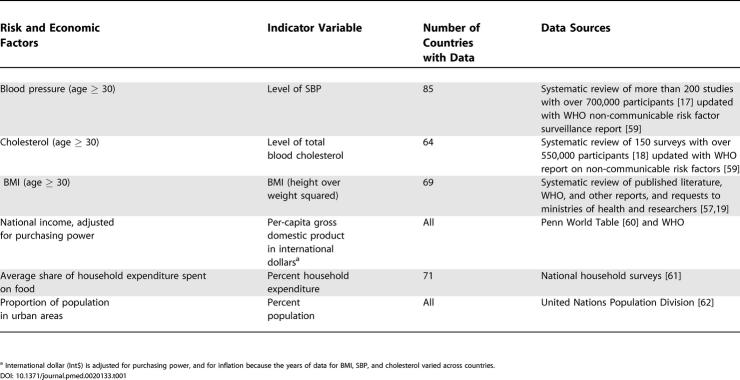
Risk and Socioeconomic Variables Used in the Analysis

^a^ International dollar (Int$) is adjusted for purchasing power, and for inflation because the years of data for BMI, SBP, and cholesterol varied across countries.

### Statistical Analysis

A local regression model was used to estimate the income–risk relationships [20]. A local regression model estimates the association across income levels without assuming a parametric model. Rather, the data determine the fitted curve. This is a preferable approach when there is no theoretical model for the shape of the association.

## Results

**Figure 2:**
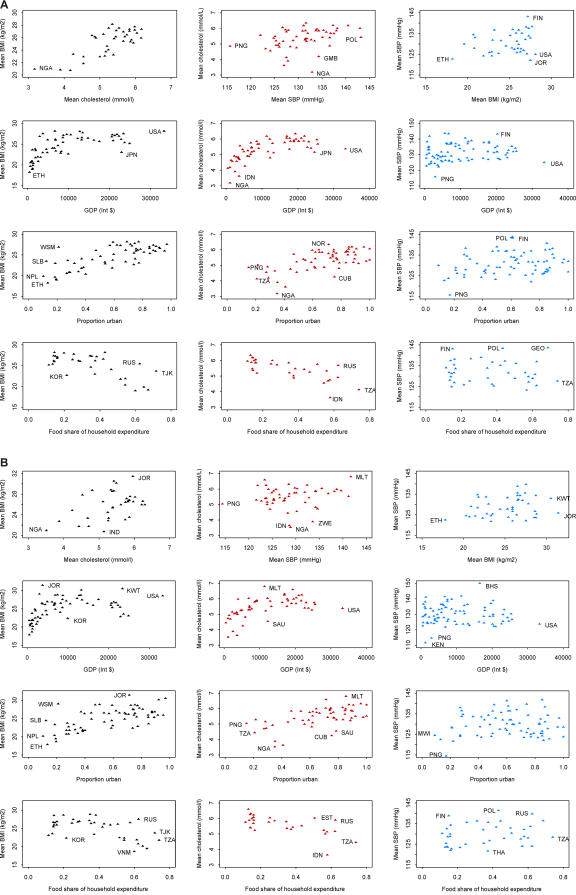
Pair-Wise Relationships of Mean Population BMI, SBP, and Total Cholesterol with National Income, Share of Household Expenditure Spent on Food, and Proportion of Population in Urban Areas Data for (A) males and (B) females are shown. National income is measured as per-capita gross domestic product (GDP). BHS, Bahamas; CUB, Cuba; EST, Estonia; ETH, Ethiopia; FIN, Finland; GEO, Georigia; GMB, Gambia; IDN, Indonesia; JOR, Jordan; JPN, Japan; KEN, Kenya; KOR, Korea; KWT, Kuwait; MLT, Malta; MWI, Malawi; NGA, Nigeria; NOR, Norway; NPL, Nepal; PNG, Papua New Guinea; POL, Poland; RUS, Russian Federation; SAU, Saudi Arabia; SLB, Solomon Islands; THA, Thailand; TJK, Tajikistan; TZA, Tanzania; USA, United States; VNM, Viet Nam; WSM, Samoa; ZWE, Zimbabwe.

**Figure 3:**
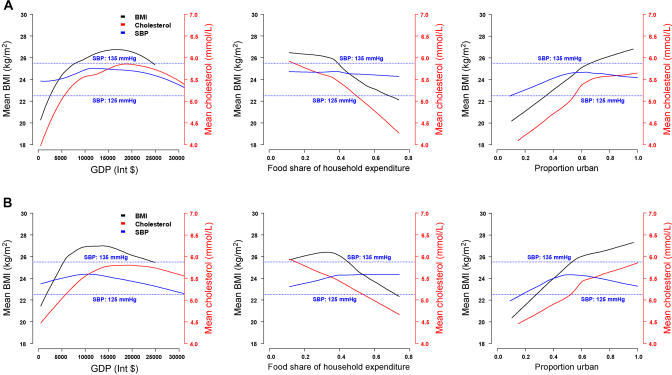
Relationship of Mean Population BMI, SBP, and Total Cholesterol with Average National Income, Food Share of Household Expenditure, and Proportion of Population in Urban Areas Relationships were estimated using local regression models applied to the data in Figure 2. Results for (A) males and (B) females are shown. National income was measured as gross domestic product (GDP). The following outlier countries were dropped (see also Results): United States for males and females in the income–BMI relationship, and Russian Federation and Tajikistan for males and females in the food share of household expenditure–BMI relationship.

When considered in relation to national income, mean population BMI and cholesterol increased, then flattened, and eventually declined (Figures 2 and 3). Mean BMI increased most rapidly until a national income of about I$5,000 (see Table S1 for national incomes of individual countries) and peaked at about I$12,500 for females and I$17,000 for males. Cholesterol's point of inflection and peak (about I$8,000 and I$18,000, respectively) were at higher income levels than those for BMI. The BMI decline relative to the peak at high incomes was larger for females than for males. The lower mean female BMI at high-income levels (except in the United States where the female BMI was 28.7 kg/m^2^) is consistent with the evidence on declining female BMI in some high-income countries (e.g., Japan [21]) over time, and with the inverse relationship between social class (as measured by education) and female obesity within upper-middle-income countries [11].

Figures 2 and 3 show that in countries where food is a relatively small proportion of total household expenditure (less than 30%–40%) (i.e., little or no constraints), there was little or no relationship between this factor and mean BMI. Where food is a larger proportion of household expenditure (more than 30%–40%), there was an inverse relationship between mean BMI and the proportion of household expenditure spent on food. Exceptions to this pattern were the Russian Federation and Tajikistan, with a large food share of household expenditure but with relatively high BMI. The departure from the overall pattern by these two countries may reflect the economic consequences of the collapse of the former Soviet Union, which have forced households to devote a large share of their expenditure to food without changing their life style determinants of weight (i.e., diet and physical activity). The relationship between the food share of household expenditure and mean blood cholesterol was similar to that for BMI, but, as in the relationship with income, the flattening of the relationship occurred later (i.e., at lower food shares) for cholesterol than for BMI. The rapid rise in mean population BMI with increasing income indicates higher consumption of energy as food purchase constraints at very low income levels are removed. That cholesterol increases more slowly and continues at higher income levels and lower food share of income than BMI may indicate that additional income is first used to increase caloric intake, followed by dietary change beyond higher calories (e.g., switch from unsaturated vegetable fats and oils to animal fat [22]) as income rises further.

The proportion of the population in urban areas was positively correlated with mean BMI and cholesterol (Figures 2 and 3). The relationships of mean BMI and cholesterol with urbanization also showed some flattening, but this was less noticeable than seen in relation to income, and occurred in countries with more than 60% of the population in urban areas. Urban living—which alters transportation and occupational patterns as well as access to various foods—may affect nutrition and activity, and increase population BMI and cholesterol, over and above its impacts mediated through income. Once a country is primarily urbanized, its infrastructure development reduces the urban–rural differences in access to food and technology [23], leading to the observed flattening of the curve at high urbanization levels. Exceptions to the relationship between cholesterol and urbanization were Indonesia and Nigeria (proportion urban 40% or more, but low cholesterol levels of 3.2–3.6 mmol/l). Both countries have large populations and, despite the formation of urban centers, still have predominantly agricultural economies and associated lifestyles.

At the aggregate level, the relationships of mean BMI and cholesterol with each other or with the economic variables were qualitatively different from those of mean SBP (Figures 2 and 3). Mean population SBP was either not correlated or only very weakly correlated with cholesterol, BMI, and the economic variables. High SBP levels were observed in some developing countries (e.g., Gambia, Ghana, and Nigeria) that had low mean cholesterol or BMI. Similarly, mean population SBP varied by 20 mm Hg in the countries of Western Europe, which have relatively similar income.

Individual-level epidemiological studies have established a relationship between BMI and blood pressure [24,25]. Blood pressure is, however, affected by other aspects of diet, including salt and fruits and vegetables [26–30] that may vary across populations independently of BMI, based on cultural and environmental factors such as food preservation techniques and subsistence versus commercial access to agricultural products. Even in within-country studies, the relationships between blood pressure and wealth or the relative levels of rural and urban blood pressure have varied, and even reversed, in different countries [31–37].

Multi-variable regression analysis (results not shown) confirmed the absence of a relationship between blood pressure and the economic variables considered (the coefficients for SBP were not statistically significant). BMI and cholesterol both increased significantly with income and as the share of expenditure used for food declined, although the size of the association was attenuated after adjustment for other factors. Countries with a larger urban population had higher BMI even after adjustment for their income.

## Discussion

Cardiovascular diseases have multiple well-established behavioral, environmental, and physiological determinants. Less well-established are the patterns of these risks at the population level in relation to one another and to economic variables such as income. Knowledge of these patterns is necessary, however, for better design of long-term policies and interventions that aim to reduce multiple risks that affect a common set of diseases in different populations, and for assessing the global health inequality dimensions of cardiovascular disease risks. Using data from over 100 countries, we found that BMI increased rapidly, then flattened, and eventually declined with increasing national income. Cholesterol had a similar, but more delayed, relationship with national income. Mean population blood pressure was not correlated or only weakly correlated with the economic factors considered, or with cholesterol and BMI. The rapid rise in BMI with income, and the lack of a relationship for blood pressure, illustrate that, currently, major transformations in patterns of cardiovascular risks occur at much earlier stages of economic development than implied by the “diseases of affluence” paradigm. If current income–risk relationships observed in Figures 2 and 3 hold as economies grow, rapidly increasing BMI, coupled with the presence of elevated blood pressure at all income levels, will increasingly concentrate two major cardiovascular risk factors (blood pressure and obesity) in populations with currently low income levels, and all three risks in currently middle-income countries.

**Figure 4:**
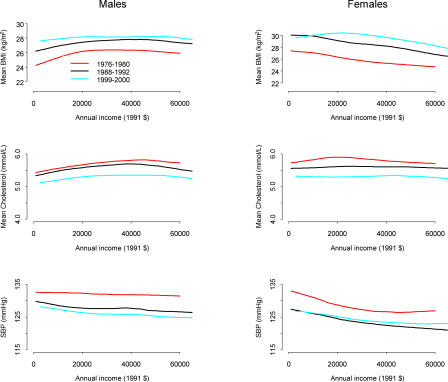
Shifting Relationships of BMI, SBP, and Total Cholesterol with Income in the United States, Estimated Using Local Regression Data are from the National Health and Examination Survey, 1976–1980, 1988–1992, and 1999–2000.

The current income–risk relationships shown in Figures 2 and 3, however, only partially illustrate the potential future magnitude and global distribution of cardiovascular risks. There are reasons to suspect that the cross-sectional relationships shown in Figures 2 and 3 may be changing over time, with important implications for the epidemiological transition. Longitudinal data from the United States and a small number of other countries show an upward shift in the entire income–BMI relationship (Figure 4). At the same time, a downward shift in the relationship between blood pressure and cholesterol with income occurred during the 1980s, followed by stabilization or a slight increase in the 1990s for blood pressure, and continued decline for cholesterol (Figure 4) [38,39]. Definitive explanations for these trends are not available. Possible reasons for BMI increase include systematic changes in diet and physical activity due to increased access to private transportation, television, and manufactured/packaged foods as a result of technological change, urbanization, and organization of work [40,41]. For blood pressure and cholesterol, possible contributors to the initial decline include changes in diet (increased access to fresh fruits and vegetables, and lower salt intake) and use of pharmacological interventions (anti-hypertensives and statins); increasing obesity may have been the obstacle to further blood pressure decline. If a similar upward shift in the income–BMI relationship occurs globally, overweight and obesity will play an even larger role in disease burden in developing countries, because these countries will be on an even higher income trajectory of BMI than shown in Figure 3. As interventions for blood pressure and cholesterol are adopted, and exposure to these risks thus is lowered, in high-income societies, the three risk factors will become increasingly concentrated in low-income and middle-income nations relative to high-income countries.

In additional to nutritional risks, tobacco smoking is also an important risk factor for cardiovascular diseases. Currently, an estimated 930 million of the world's 1.1 billion smokers live in the developing world [42]. Tobacco smoking increased among men, followed by women, in industrialized nations in the last century, and has subsequently declined in some nations (e.g., Canada, the United States, and the United Kingdom) [43]. Descriptive models based on historical patterns in the industrialized world [44] would predict a decline in male smoking and an increase in female smoking in the developing world over the coming decades. However, there have been major recent transformations in global tobacco trade, marketing, and regulatory control. As a result, tobacco consumption among men and women in most nations is primarily determined by opposing industry efforts and tobacco control measures [45], and by the socio-cultural context, rather than national income. Sex-specific data on tobacco consumption are very rare; the available data on smoking prevalence [42,46] indicate little relationship with national income.

The observed patterns of multiple nutritional risk factors also have implications for risk factor measurement and surveillance. Ideally, analysis of cardiovascular disease risks should use longitudinal data to assess the effects of socioeconomic factors on nutrition and nutritional risk factors both within and across populations. Detailed longitudinal data on nutrition (including size and composition of diet and frequency of eating) and physical activity [47,48], or on associated indicator risk factors like blood pressure and cholesterol, are very scarce, especially in developing countries. Partly because of data limitations, the emphasis of global nutritional surveillance has been on dietary composition using aggregate date sources (e.g., the United Nations Food and Agriculture Organization's FAOSTAT; http://apps.fao.org/). Analyses of these aggregate data sources indicate that a shift towards “Western diets” high in saturated fat and sugar and low in fiber is occurring [2,41]. Food composition and total caloric intake, although interrelated [49,50], would have distinct influences on intermediate risks like BMI and cholesterol. The observed rapid BMI rise with national income indicates that dietary composition, which dominates global nutritional surveillance, should also be explicitly considered in relation to other determinants of weight such as frequency of eating and physical activity in leisure, occupation, and transportation domains [51]. Better data on food consumption and physical activity in turn require developing low-cost and valid instruments for large-scale population health surveys.

Multi-risk assessment also demonstrates intervention needs and options in societies at different income levels. The observed rapid BMI increase with national income indicates that preventing obesity, which may be more effective than reacting after it has occurred [52], should be a priority during economic growth and urbanization. Overweight and obesity are also important because they cause a number of non-cardiovascular outcomes (cancers, diabetes, and osteoarthritis) which cannot be addressed by reducing risk factors such as blood pressure and cholesterol. Current intervention options for obesity in principle include those that reduce caloric intake (e.g., agriculture and food policy and pricing) and those that increase energy expenditure (e.g., urban design and transportation) [14,40]. There is currently limited evidence on the community effectiveness of these interventions [52,53]. This limitation highlights the need for research on design and evaluation of interventions for obesity—which in turn requires better data on the relative contributions of nutritional and physical activity to the current trends in weight gain—and on the obesity implications of policies and programs in sectors like transportation and agriculture. Multi-risk assessment also demonstrates that personal and population-level interventions for blood pressure and cholesterol (e.g., salt awareness and regulation, and pharmacological interventions) should be pursued together with attempts to curb or reduce obesity, because these interventions are effective in reducing the cardiovascular consequences of nutritional risk factors [54,55].

The division between the diseases of poverty and affluence has provided a convenient tool for targeting policies towards risks such as undernutrition that affect the poor [56]. Demographic and technological change, however, are increasingly modifying the income patterns of cardiovascular risk factors and shifting their burden to the developing world. As a result, low-income and middle-income countries increasingly face the double burden of infectious disease and cardiovascular risk factors. Unless the research and intervention needs described earlier are pursued, this will create a world in which all major diseases are the diseases of the poor.

## Supporting Information

Table S1 Countries Used in the Analysis and Data Availability (38 KB PDF).

**Patient Summary**

**Background**

The pattern of cardiovascular diseases is influenced by many lifestyle factors such as diet, physical activity, work and leisure, and smoking. The effects of these factors are partly mediated through intermediate risk factors like overweight and obesity, blood pressure, and cholesterol. As societies grow richer, the patterns of risk-factor exposure change. Understanding these changes is important for health policies and interventions.

**What Did the Researchers Do?**

They looked at cardiovascular disease risks such as being overweight or obese, systolic blood pressure, and total cholesterol, and related them to national income, food purchase constraints, and urbanization. Body mass index (BMI) and cholesterol increased as national income increased, then flattened, and eventually declined. BMI increased until an income of about I$12,500 (international dollars—a currency adjusted for differences in prices in different countries) for females and I$17,000 for males, then flattened, and eventually declined. Cholesterol showed the same pattern, but with some delay. As the proportion of household spending devoted to food decreased (i.e., due to changes in income or the price of food), BMI and cholesterol levels increased. Also, as more people lived in cities, the population's BMI went up.

**What Do These Findings Mean?**

These findings are from a comparison of countries at one point in time, so they may only partly predict the path of individual countries over time. What they do suggest is that cardiovascular disease risks will increasingly be concentrated in low-income and middle-income countries. Therefore, preventing obesity should now be considered a priority even in these countries, along with measures to control blood pressure, cholesterol, and tobacco use.

**Where Can I Get More Information?**

The World Health Organization has a Web site on nutrition: http://www.who.int/nut/

MedlinePlus has a selection of topics on obesity: http://www.nlm.nih.gov/medlineplus/obesity.html

## Abbreviations

BMI: body mass index
SBP: systolic blood pressure
WHO: World Health Organization
