# Supplementary material for: Rethinking the “Diseases of Affluence” Paradigm: Global Patterns of Nutritional Risks in Relation to Economic Development
Source: PLoS Med. 2005 May 3;2(5):e133. doi: 10.1371/journal.pmed.0020133 (PMC1088287; doi:10.1371/journal.pmed.0020133)
Supplement: Supplementary file 1 [file pmed.0020133.st001.pdf]

Supplementary Online Material

Table S1: Countries used in the analysis and data availability for systolic blood pressure (SBP), cholesterol, body mass index (BMI), and food share of household expenditure. Data on national income and proportion of population in urban areas were available for all countries.

**Country** [1, 2]	**Code**	**2000 income (Int$)** [3]	**SBP** [4, 5]	**Cholesterol** [4, 5]	**BMI** [4, 5]	**Food share of expenditure**
African region						
Burundi	BDI	523				✓
Cameroon	CMR	2,042	✓		✓	
Democratic Republic of the Congo	ZAR	315	✓			
Ethiopia	ETH	635	✓		✓	
Gambia	GMB	1,217	✓	✓	✓	
Ghana	GHA	1,351	✓	✓	✓ (F)	✓
Kenya	KEN	1,244	✓		✓ (F)	
Lesotho	LSO	1,592	✓			
Liberia	LBR	2477	✓			
Malawi	MWI	784	✓		✓	
Mali	MLI	969			✓	
Mauritius	MUS	13,932	✓			✓
Namibia	NAM	4,381				✓
Nigeria	NGA	707	✓	✓	✓	
Senegal	SEN	1,622	✓		✓	✓
Seychelles	SYC	10,241	✓	✓	✓	
Sierra Leone	SLE	534	✓			
South Africa	ZAF	7,541	✓	✓	✓	✓
United Republic of Tanzania	TZA	482	✓	✓	✓ (F)	✓
Zambia	ZMB	892				✓
Zimbabwe	ZWE	2,486	✓	✓	✓	
Region of the Americas						
Argentina	ARG	11,006	✓		✓	✓
Bahamas	BHS	13,134	✓			
Barbados	BRB	16,415	✓		✓	
Belize	BLZ	6,591	✓			
Brazil	BRA	7,190	✓	✓ (M)	✓	✓
Canada	CAN	26,904	✓	✓	✓	✓
Chile	CHL	9,926	✓	✓		
Colombia	COL	5,383	✓	✓ (M)		✓
Costa Rica	CRI	5,870				✓
Cuba	CUB	2,499		✓	✓	
Dominican Republic	DOM	5,270	✓	✓		
Ecuador	ECU	3,468	✓			
Guatemala	GTM	3,914			✓ (F)	
Guyana	GUY	3,520				✓
Haiti	HTI	1,021	✓			
Jamaica	JAM	3,693	✓	✓		✓
Mexico	MEX	8,762	✓	✓	✓	✓

Nicaragua	NIC	1,767				✓
Panama	PAN	6,066				✓
Paraguay	PRY	4,684	✓		✓	✓
Peru	PER	4,589			✓ (F)	✓
Saint Lucia	LCA	6,330	✓		✓	
Trinidad and Tobago	TTO	11,175	✓			
United States of America	USA	33,293	✓	✓	✓	✓
Uruguay	URY	9,622	✓	✓		
Venezuela (Bolivarian Republic of)	VEN	6,420	✓	✓		
Eastern Mediterranean region						
Bahrain	BHR	12,685		✓	✓	
Djibouti	DJI	1,158				✓
Egypt	EGY	4,184	✓	✓	✓ (F)	✓
Iran (Islamic Republic of)	IRN	5,995	✓	✓	✓	
Jordan	JOR	3,895	✓	✓	✓	
Kuwait	KWT	14,278	✓	✓	✓	
Lebanon	LBN	5,786			✓	✓
Morocco	MAR	3,717				✓
Pakistan	PAK	2,008			✓	✓
Saudi Arabia	SAU	11,764	✓	✓	✓	
Tunisia	TUN	6,776	✓	✓		✓
United Arab Emirates	ARE	20,468	✓	✓ (M)	✓	
Yemen	YEM	817				✓
European region						
Albania	ALB	3,346			✓	✓
Andorra	AND	22,573				
Austria	AUT	23,676	✓	✓		
Azerbaijan	AZE	2,798				✓
Belgium	BEL	23,781	✓	✓	✓	✓
Bulgaria	BGR	5,785				✓
Croatia	HRV	8,526				✓
Czech Republic	CZE	13,666	✓	✓	✓	✓
Denmark	DNK	26,608	✓	✓	✓	✓
Estonia	EST	9,589	✓	✓		✓
Finland	FIN	23,792	✓	✓	✓	✓
France	FRA	22,358	✓	✓		✓
Georgia	GEO	4,906	✓			✓
Germany	GER	22,856	✓	✓	✓	✓
Greece	GRC	14,614		✓	✓	✓
Hungary	HUN	10,439	✓	✓	✓	✓
Iceland	ISL	24,777	✓	✓	✓	✓
Ireland	IRL	26,381	✓			
Israel	ISR	16,954	✓	✓		✓
Italy	ITA	21,780	✓	✓		
Kazakhstan	KAZ	7,391				✓
Kyrgyzstan	KGZ	3,015				✓
Latvia	LVA	7,653			✓	✓

Lithuania	LTU	7,253	✓	✓ (M)	✓	✓
Malta	MLT	8,353	✓	✓	✓	
Netherlands	NLD	24,313	✓	✓	✓	
Norway	NOR	27,060	✓	✓		✓
Poland	POL	9,217	✓	✓		✓
Portugal	PRT	15,923		✓	✓	✓
Romania	ROM	4,285	✓	✓	✓	✓
Russian Federation	RUS	8,009	✓	✓	✓	✓
Slovakia	SVK	11,410			✓	✓
Slovenia	SVN	15,745	✓			✓
Spain	ESP	18,047	✓	✓	✓	✓
Sweden	SWE	23,635	✓	✓		✓
Switzerland	CHE	26,414	✓	✓	✓	✓
Tajikistan	TJK	1,321			✓	✓
Turkey	TUR	6,832	✓	✓	✓	
Ukraine	UKR	4,622				✓
United Kingdom	GBR	22,190	✓	✓	✓	✓
Uzbekistan	UZB	2,179	✓		✓	
Yugoslavia	YUG	5,239	✓			
South east Asian region						
Bangladesh	BGD	1,684			✓	✓
India	IND	2,479	✓	✓	✓ (F)	✓
Indonesia	IDN	3,642	✓	✓		✓
Nepal	NPL	1,459			✓	✓
Sri Lanka	LKA	3,300	✓			✓
Thailand	THA	6,857	✓	✓	✓	✓
Western Pacific region						
Australia	AUS	25,559	✓	✓	✓	
Cambodia	KHM	1,245				✓
China	CHN	3,747	✓	✓	✓	
Japan	JPN	24,675	✓	✓	✓	
Kiribati	KIR	1,638		✓		
Malaysia	MYS	9,919			✓	
Mongolia	MNG	1,718	✓			
Nauru	NRU	11,154	✓	✓		
New Zealand	NZL	1,8816	✓	✓		
Papua New Guinea	PNG	3,092	✓	✓		
Philippines	PHL	3,425		✓ (M)	✓	✓
Republic of Korea	KOR	15,876	✓		✓ (F)	✓
Samoa	WSM	2,999			✓	
Singapore	SGP	23,765	✓	✓		
Solomon Islands	SLB	2,082			✓	
Viet Nam	VNM	2,241			✓	✓

[1] Blood pressure and/or cholesterol data for Cameroon, Colombia, Ghana, Mauritius, Senegal, Saint Lucia, Venezuela, Tunisia, Greece, and Haiti were excluded from the analysis because the populations of these countries live primarily in rural areas but the data were from urban populations and/or the study sample sizes were very small.

[2] BMI data for Paraguay, Albania, Romania, and Malta were excluded from the analysis because the populations of these countries live primarily in rural areas but the data were from urban populations and/or the study sample sizes were very small.

[5] Income data (for 2000 and for the analyses in Figures 2 and 3) were from the Penn World Table [Heston, 2002 #318] and from the Global Programme on Evidence for Health Policy, WHO for countries for which Penn World Table did not provide estimates. Because income data for some country-years of interest were not available, the following substitutions were made in Figures 2 and 3: BMI: Cuba 1985 for 1982, Bahrain 1996 for 2000, Kuwait 1996 for 1993, Saudi Arabia 1996 for 1992, Malta 1994 for 1984, Viet Nam 1989 for 1985. Data for Samoa, Solomon islands, United Arab Emirates were from estimates by the Global Programme on Evidence for Health Policy, WHO. Cholesterol: Zimbabwe 2000 for 2001, Cuba 1996 for 2000, Jamaica 2000 for 2001, Bahrain 1996 for 2000, Jordan 2000 for 2001, Saudi Arabia 1996 for 1991, Malta 1994 for 1984, Estonia 1992 for 1991, Republic of Moldova 1995 for 1992, Russian federation 1991 for 1989, Indonesia 2000 for 2001. Data for Kiribati and United Arab Emirates were from estimates by the Global Programme on Evidence for Health Policy, WHO. Blood pressure: Bahamas 1996 for 1988, Haiti 1998 for 2000, Saudi Arabia 1996 for 1995, Malta 1994 for 1989, Slovenia 1990 for 1988, Turkey 200 for 2002, Uzbekistan 1996 for 1997, Mongolia 1996 for 1999. Data for Liberia, United Arab Emirates, Yugoslavia, and Nauru were from estimates by the Global Programme on Evidence for Health Policy, WHO.

[4] Data were available for both females and males unless indicated.

[5] The year of survey was not reported in some studies. In such cases, the year that the study was first published was used. This occurred for one risk factor in Ghana, Mauritius, Senegal, Zimbabwe, Barbados, Brazil, Chile, Jamaica, Belize, Venezuela, Bahrain, Jordan, Greece, Denmark, Georgia, Lithuania, Portugal, and Philippines.
